# Association of *ARNTL* and *PER1* genes with Parkinson's disease: a case-control study of Han Chinese

**DOI:** 10.1038/srep15891

**Published:** 2015-10-28

**Authors:** Zhuqin Gu, BinBin Wang, Yong-Biao Zhang, Hui Ding, Yanli Zhang, Jun Yu, Mingliang Gu, Piu Chan, Yanning Cai

**Affiliations:** 1 Department of Neurobiology, Xuanwu Hospital of Capital Medical University, Key Laboratory for Neurodegenerative Diseases of the Ministry of Education, Beijing 100053, P.R. China; 2 CAS Key Laboratory of Genome Sciences and Information, Beijing Institute of Genomics, Chinese Academy of Sciences, Beijing 100101, P.R. China; 3 National Research Institute for Family Planning, Beijing 100081, P.R. China; 4 Department of Neurology, Xuanwu Hospital of Capital Medical University, Key Laboratory for Neurodegenerative Diseases of the Ministry of Education, Beijing 100053, P.R. China

## Abstract

Circadian disruptions may result in sleep problems, oxidative stress and an altered inflammatory response. These symptoms may contribute to PD pathogenesis, despite a lack of direct experimental evidence supporting this relationship. Clock genes are essential to drive and maintain circadian rhythm. To elucidate the possible role of circadian disruptions in PD, we investigated 132 tag variants in eight clock genes. We genotyped these tags within 1,394 Chinese cases and 1,342 controls using Illumina GoldenGate chips. We discovered that SNPs in *ARNTL* (rs900147, *P* = 3.33 × 10^−5^, OR = 0.80) and *PER1* (rs2253820, *P* = 5.30 × 10^−6^, OR = 1.31) genes are significantly associated with PD risk. Moreover, the positive association of the *ARNTL* rs900147 variant was more robust in tremor dominant (TD) (*P* = 3.44 × 10^−4^) than postural instability and gait difficulty (PIGD) cases (*P* = 6.06 × 10^−2^). The association of the *PER1* rs2253820 variant was more robust in PIGD (*P* = 5.42 × 10^−5^) than TD cases (*P* = 4.2 × 10^−2^). Haplotype analysis also showed that *ARNTL* and *PER1* were associated with PD. Imputation analysis identified more SNPs within *ARNTL* and *PER1* associated with PD, some of which may affect gene expression through altering the transcription factor binding site. In summary, our findings suggest that genetic polymorphisms in *ARNTL* and *PER1* genes, as well as circadian disruptions, may contribute to PD pathogenesis.

Circadian rhythms are physiological and behavioural cycles generated by an endogenous biological clock. Circadian disruption has global adverse effects on human health and is speculated to exacerbate a variety of neurodegenerative diseases^1^. Parkinson's disease (PD) is the second most common neurodegenerative disorder, affecting more than 4 million people worldwide. PD patients show dopaminergic neuron degeneration in the substantia nigra, which leads to a complex motor disorder featuring bradykinesia, tremor, rigidity and postural instability. The aetiology of PD is complicated, and it is believed that a combination of insults contribute to the pathogenesis of the disease. These include ageing, mitochondria dysfunction, oxidative stress, axon energy deficiency, altered protein handling and inflammatory changes^2^,^3^,^4^,^5^.

Circadian disruptions have been well documented in patients with Parkinson’s disease (PD)^6^. Accumulating evidence indicates that these perturbations can occur at very early stages of the disease and may precede motor symptoms. For example, profound circadian abnormalities have been observed in patients with newly diagnosed PD^7^. Furthermore, altered circadian periodicity and locomotor activity have been demonstrated in PD animals without obvious neuronal loss or movement symptoms^8^,^9^. Indeed, because circadian disruptions may result in sleep problems, oxidative stress and an altered inflammatory response, they could also contribute to PD pathogenesis^10^,^11^, despite a lack of direct experimental evidence demonstrating these effects.

In the last decade, the intracellular molecular machinery underlying circadian rhythms has become increasingly clear. Interplay of autoregulatory transcriptional and translational feedback loops consisting of a set of key clock genes, including *NPAS2*, *CLOCK*, *RORB*, *ARNTL*, *CRY1*, *CRY2*, *PER1* and *NR1D1*, drive and maintain circadian oscillations^12^. Animals without functional clock genes show substantial changes in circadian rhythm. For example, *ARNTL* knockout mice lose their rhythm in constant darkness^13^. Mice with mutant *PER1* present rhythmicity almost 1 hour shorter than wild-type littermates^14^.

To test whether polymorphisms in genes involved in circadian disruptions are associated with susceptibility to PD, 132 tag single-nucleotide polymorphisms (SNPs) in 8 key clock genes were retrospectively examined in 1,440 PD patients and 1,400 control subjects. To our knowledge, this is the first study exploring the relationship between genetic polymorphisms of the circadian clock and PD risk.

## Results

### Demographic and clinical characteristics

Demographic and clinical characteristics of the PD cohort and control subjects are provided in Table 1. All patients were sporadic late-onset PD with an onset age above 45 years. Approximately 59.5% of control subjects were male, with a mean age of 66.5 ± 7.2 years (Table 1). Among these subjects, 46 patients and 58 controls were excluded from the data analysis due to unsuccessful genotyping. A total of 1,394 PD patients and 1,342 control subjects were genotyped successfully.

Tremor/postural instability and gait difficulty (PIGD) (t/p) scores were calculated in 1,253 patients. Among these patients, 395 were classified as tremor dominant (TD) subtype, with a t/p score ≥1.5; 666 patients were classified as PIGD phenotype, with a t/p score ≤1; and the remaining 192 patients were categorised as intermediate (IND). The control subjects were matched to cases with respect to sex, age, and ethnic origin.

### Quality control

**Figure 1:**
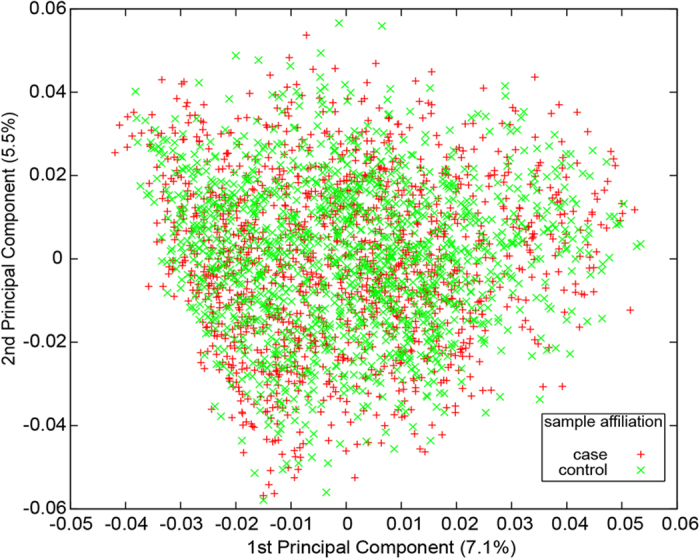
Principal component analysis on 1,394 cases and 1,342 controls using 125 QC-passed SNPs. We plotted the first and second principal components to show the range of the studied samples. The percentages of variance accounted for by the two components are indicated in brackets. The sample affiliation is shown by different symbols in red (cases) or green (controls).

For the eight key clock genes, 128 of 132 SNPs were accurately clustered, and 125 SNPs were in Hardy-Weinberg equilibrium in the control group. A two-dimensional scaling plot generated from the two highest eigenvectors showed that the cases and controls were uniformly mixed, indicating no population stratification (Fig. 1). In the end, 125 SNPs and 2,736 individuals (1,394 cases and 1,342 controls; 1,712 males and 1,168 females), with a successful genotyping rate of 99%, passed quality control and were used for association studies.

### Overall data synthesis

We observed that ≥1 SNPs with a *P*-value < 0.05 for *NPAS2*, *CLOCK*, *RORB*, *ARNTL*, *PER1* and *NR1D1* genes, but none for *CRY2* and *CRY1* genes (Table 2, Supplementary Table 1). Most of the SNPs were seen the potential association with PD under trend genetic model and additive logistic regression model. The protective per-allele odds ratio (OR) ranged from 0.80 to 0.88, and the susceptibility per-allele OR ranged from 1.17 to 1.31. After adjusting for multiple comparisons, rs900147 (*P* = 3.33 × 10^−5^; OR = 0.80; Trend model) and rs2253820 (*P* = 5.30 × 10^−6^; OR = 1.31; Trend model) within *ARNTL* and *PER1*, respectively, passed Bonferroni correction. No SNPs within other genes had significant Bonferroni-corrected *P*-values (*P*-value < 0.05). Gene-gene interactions were evaluated for each pair of SNPs, and none of them satisfied the Bonferroni-like correction developed by Emily *et al.*^15^.

**Figure 2:**
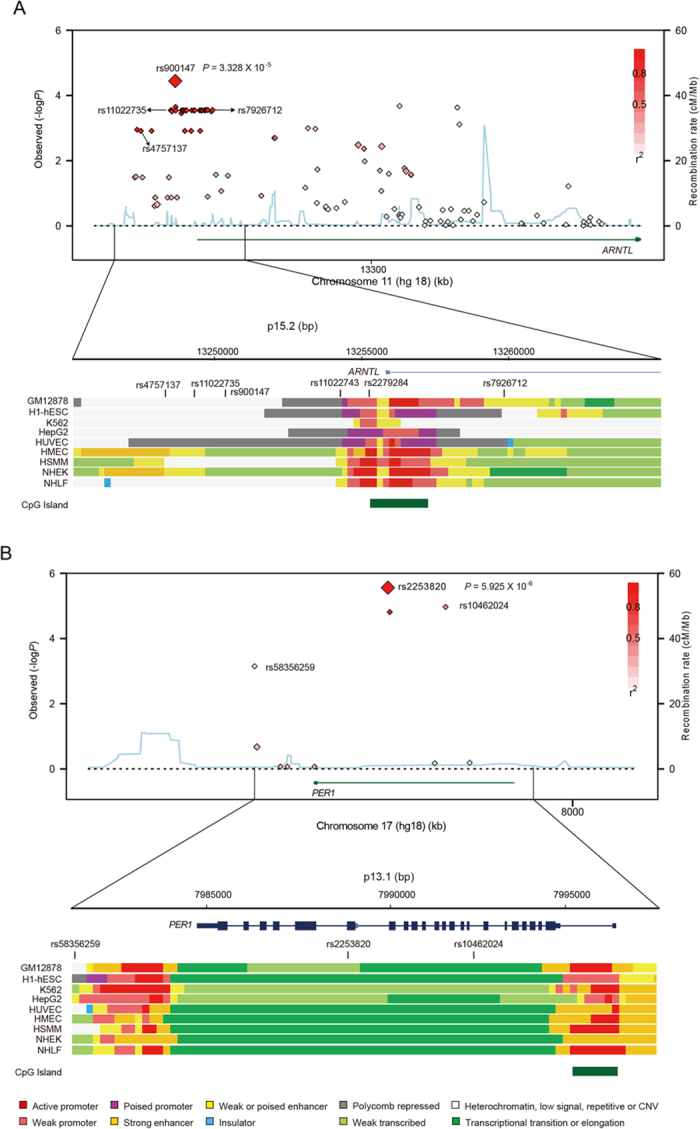
Regional plots of association results, recombination rates and chromatin state segmentation tracks for *ARNTL* and *PER1*. (**A**,**B**) Results for *ARNTL* (**A**) and *PER1* (**B**), respectively. Plots show the association results of both genotyped and imputed SNPs in the studied samples and their recombination rates. log_10_ *P*-values (y axes) of the SNPs are shown according to their chromosomal positions (x axes). The lead SNP in each combined analysis is shown as a large diamond and labelled by its rsID and *P*-values. The colour intensity of each symbol reflects the extent of LD with the lead SNP, white (r^2^ = 0) through to dark red (r^2^ = 1.0). Genetic recombination rates, estimated using CHB+JPT samples from 1,000 genomes, are shown with a light blue line. Physical positions are based on hg18 of the human genome. The relative positions of genes and transcripts mapping to the region of association are also shown. Genes have been redrawn to show their relative positions. Below each plot is a diagram of the exons and introns of the genes and SNPs of interest. The chromatin state segmentation track (ChromHMM) for 9 studied cell lines was derived from the ENCODE project (UCSC genome browser). The CpG island is shown at the bottom of each plot.

In the *ARNTL* gene, another 5 SNPs showed marginal associations with PD (Table 2). These SNPs had a moderate correlation (r^2^ > 0.4) with rs900147, except for rs1562437 (*P* = 0.00133; OR = 0.81; r^2^ = 0.02), which might have an independent association with PD. We performed conditional analysis on SNPs in *ARNTL* with rs900147 as a covariate, and we found that rs1562437 (conditional *P* = 0.00494) was independently associated with PD. In the *PER1* gene, only rs2253820 showed a significant association with PD among 4 genotyped tagging SNPs (Fig. 2B). Conditional analysis showed that the 3 remaining *PER1* tags were not independently associated with PD. Therefore, we found another SNP of rs1562437 within *ARNTL* that was independently associated with PD.

### Association between SNPs and PD subtypes

PD patients were categorised into three subtypes according to their t/p scores, including tremor dominant (TD), postural instability and gait difficulties dominant (PIGD) or intermediate (IND). Different patterns were observed among PD subtypes (Table 3). For PIGD subtype vs. controls, rs2253820 showed a strong association (*P* = 5.42 × 10^−5^, Bonferroni *P* = 6.77 × 10^−3^), and the OR improved from 1.31 (all PD) to 1.42 (PIGD PD). In contrast, rs900147 only showed a borderline significant association (*P* = 6.06 × 10^−2^) with the PIGD subtype and failed to survive a Bonferroni correction. For TD vs. controls, rs900147 showed a strong association (*P* = 3.44 × 10^−4^, Bonferroni *P* = 4.30 × 10^−2^), and the OR improved from 0.80 (all PD) to 0.78 (TD PD). In contrast, rs2253820 showed a weak association (*P* = 4.20 × 10^−2^) with the TD subtype and failed to survive a Bonferroni correction. Subgroup analysis showed that rs2253820 may be associated with the PIGD subtype, and rs900147 may be specifically associated with the TD subtype.

### Imputation and functional elements prediction

Using IPMUTE2, we imputed the genotype of untyped SNPs in *ARNTL* and *PER1* based on typed SNPs and 1,000 Genomes phase 1 integrated variant set. Numerous imputed SNPs showed significant associations with PD (Fig. 2), including rs11022743 (*P* = 1.28 × 10^−3^), rs2279284 (*P* = 1.28 × 10^−3^) and rs4757137 (*P* = 1.26 × 10^−3^) in *ARNTL* and rs58356259 (*P* = 7.11 × 10^−4^) in *PER1*. Several of them were located in the enhancer or promoter region of *ARNTL* (Fig. 2). The change at rs11022743 potentially eliminates the binding site for the EVI-1 transcription factor (TFSEARCH score of 91.3), and the substitution at rs2279284 adds a putative binding site for the CdxA transcription factor (TFSEARCH score of 87.1). Imputation analysis identified more SNPs within *ARNTL* and *PER1* that were associated with PD, some of which may affect corresponding expression levels through altering transcription factor binding sites.

### Haplotype-based association study

In the *ARNTL* gene, 5 common haplotypes (allele frequency greater than 0.05) were inferred from 5 correlated SNPs surrounding rs900147. Two of these (GCGAA and AAACG) showed significant (permutation *P* < 0.05) associations with PD (Table 4). The frequency of the GCGAA haplotype was significantly higher in patients than controls [permutation *P* = 0.0182; OR = 1.18 (95% CI: 1.02–1.39)]. In contrast, the frequency of AAACG was significantly lower in patients than controls [permutation *P* = 0.0102; OR = 0.90 (95% CI: 0.79–0.98)]. In the *PER1* gene, the correlated SNPs rs2225380 and rs2304911 formed three haplotypes (Table 4). The frequency of the GA haplotype was significantly higher in patients than controls [permutation *P* = 1.00 × 10^−4^; OR = 1.21 (95% CI: 1.05–1.39)]. The frequency of the AA haplotype was significantly lower in patients than controls [permutation *P* = 1.00 × 10^−4^; OR = 0.89 (95% CI: 0.78–0.97)]. Haplotype analysis also showed that *ARNTL* and *PER1* were associated with PD.

### Linkage disequilibrium (LD) blocks

**Figure 3:**
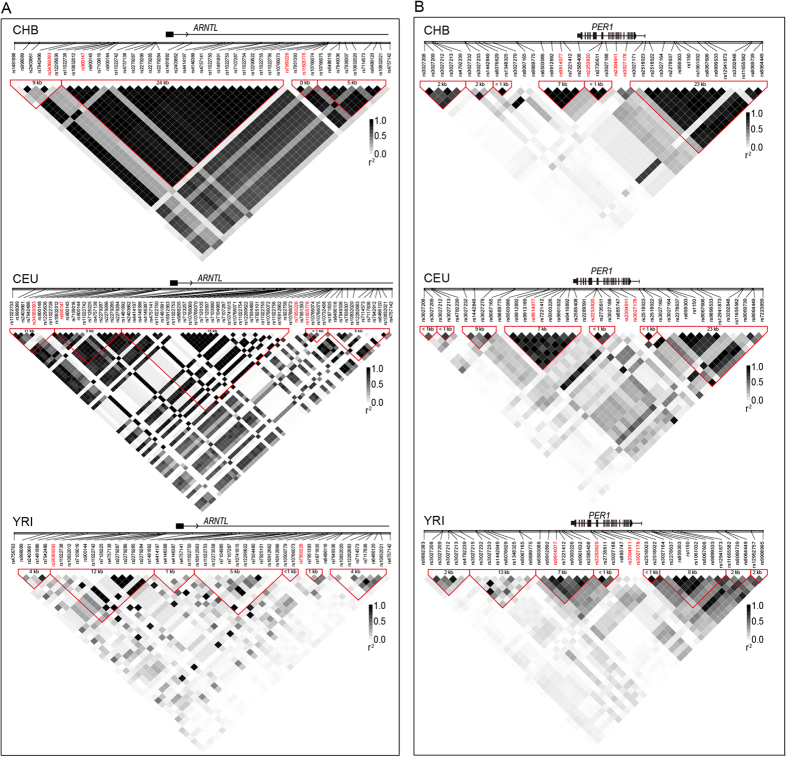
Linkage disequilibrium (LD) blocks the most significantly associated SNPs in *ARNTL* (rs990147) and *PER1* (rs2253820) in 3 HapMap populations. HapMap Phase II+III data surrounding (approximately 50 kb) rs990147 (panel **A**) and rs2253820 (panel **B**) were used to construct LD blocks of CHB (Han Chinese in Beijing, China), CEU (Utah residents with Northern and Western European ancestry from the CEPH collection) and YRI (Yoruba in Ibadan, Nigeria) using Haploview software. SNPs with minor allele frequency >0.1 were used to calculate the r^2^ and to construct the LD block. From the top to bottom of each diagram are genes and their relative positions, relative positions of SNPs on the x-axis, ID of each SNP, LD block and their size, and r^2^ values of paired SNPs, which were represented as squares with colour inside, white (r^2^ = 0) to black (r^2^ = 1.0). The relative positions of SNPs are according to their physical position on the chromosome and are labelled with vertical bars. The LD block was determined using confidence intervals (CIs) and was labelled with red triangles. The block size was determined by the distance of border SNPs and annotated in each block. Tagging SNPs are labelled in red.

We constructed LD blocks of genomic regions surrounding the most significant SNPs (rs900147 and rs2225380) using HapMap phase II+III data (Fig. 3). In the Chinese Han Beijing (CHB) population, rs900147 was found to reside in a 24-Kb block containing the promoter region and the first exon of *ARNTL*. However, in the CEU (Utah residents with Northern and Western European ancestry from the CEPH collection) and YRI (Yoruba in Ibadan, Nigeria) populations, the 24-Kb block ragged into several smaller blocks. Additionally, rs900147 resided in a block not in LD with the promoter region and first exon of *ARNTL*, as well as rs11022743 and rs2279284. This location may affect the *ARNTL* expression level. Therefore, different LD patterns in Chinese individuals should be taken into consideration for replication studies of *ARNTL* and PD in other populations.

## Discussion

We hypothesised that clock genes involved in circadian disruption may contribute to PD pathogenesis. In this hypothesis-driven case-control study, we demonstrated that genetic variants in clock genes are associated with individual susceptibility to PD. Significant associations were observed for the *ARNTL* and *PER1* genes, while nominally significant associations were found for the *NPAS2*, *CLOCK*, *RORB* and *NR1D1* genes. ORs for these associations were moderate, with the protective per-allele OR ranging from 0.80 to 0.88 and the susceptibility per-allele OR ranging from 1.17 to 1.31. The results were comparable with other well-established PD genes, such as *SNCA* (OR = 1.57; 95% CI: 1.42–1.72) and *MAPT* (OR = 1.12; 95% CI: 1.02–1.23)^16^.

This study is the first to examine the association between clock genes and PD risk. It is noteworthy that genetic variants in clock genes have been reported to be associated with susceptibility to prostate cancer, diabetes and bipolar disorders^17^,^18^,^19^. Interestingly, patients with these diseases are more likely to develop PD^20^,^21^,^22^.

Breen and Vuono reported a lack of time-dependent variation in *ARNTL* expression in PD patients compared with controls^7^. We also discovered reduced *ARNTL* expression in PD patients^23^. The precise nature of the altered *ARNTL* expression in PD needs to be investigated. However, the close interaction between the circadian and dopamine systems may provide possible explanations for this effect. On one hand, dopamine has been shown to be capable of regulating ARNTL/CLOCK heterodimer activity and the expression of a variety of clock genes^24^, possibly suggesting that dopamine deficiency directly affects the molecular clock's central component. Conversely, transcription of tyrosine hydroxylase, the rate-limiting enzyme in dopamine biosynthesis, is under the control of the molecular clock^25^. Meanwhile, daily variations of dopamine and its metabolites are also well documented^26^. The interaction between circadian and dopamine systems suggests a vicious cycle in PD involving the molecular clock. This study showed that *ARNTL* is also associated with PD at the genetic level, which is in line with expression studies and provides novel insight regarding the interaction between circadian and dopamine systems.

Our findings support evidence that circadian disruptions may contribute to PD pathogenesis. Consequently, targeting circadian dysfunction may have therapeutic potential for PD. Several approaches have been documented that strengthen circadian rhythms in humans, including bright light exposure, melatonin administration, and scheduled social cues^27^. Of note, a limited daily regimen of light exposure is likely to modulate circadian rhythms, causing progressive degeneration associated with PD^28^. Further studies are required to determine whether these approaches can slow the progression of the disease, especially in patients in the pre-motor stage.

Circadian disruptions have broad negative impacts on human health^29^. For example, circadian perturbations can lead to an increase in oxidative stress, mitochondrial dysfunction and inflammation^30^, which may accelerate neurodegeneration in PD. In addition, sleep is important for the removal of neurotoxic waste and is therefore closely related to neurodegenerative disorders^31^. Circadian disruptions and sleep problems appear to be able to exacerbate one another^7^,^32^. Circadian disruptions may accelerate PD progression via a mechanism involving sleep regulation. It is important to clarify how circadian disruptions and sleep problems jointly promote PD development.

Interestingly, the positive association of the *ARNTL* variant (rs900147) was more robust in TD cases. In contrast, the positive association of the *PER1* variant (rs2225380) was more robust in PIGD cases. Allele frequencies for rs900147 and rs2225380 did not differ significantly (*P* = 0.31 and *P* = 0.18, respectively; chi-square test) between TD (n = 395) and PIGD cases (n = 666). This finding likely occurred because the number of subjects in each PD subtype was quite limited. PD is a heterogeneous disorder, with differences existing between patients presenting with tremor versus non-tremor symptoms. Multiple single-photon emission studies indicate that these two types of motor symptoms likely arise from disturbances in different neural systems^33^. In addition, non-tremor patients experience faster disease progression and more frequent dyskinesias, gait disorders, and falls^34^. Whether *ARNTL* and *PER1* affect disease progression differently needs to be further investigated.

Clock genes were not found to be associated with PD in previous genome-wide association studies (GWAS). There are several possible reasons for a lack of an association. Firstly, PD is heterogeneous, and some PD candidate genes are only associated with a subtype of PD^35^. These genes would escape detection in an un-stratified GWAS study. Indeed, our data indicate that *ARNTL* and *PER1* are more closely associated with a particular PD subtype. Secondly, because a very large number of SNPs are examined simultaneously in GWAS, an extremely low *P*-value threshold has to be used for genome-wide significance. This may lead to the omission of some marginally associated genes. Thirdly, genetic backgrounds differ substantially among studied populations^36^. Most PD GWAS studies were carried out in Caucasian populations, while our investigation was performed in Chinese Han. Indeed, the patterns of LD blocks at *ARNTL* differed among CHB+JPT, CEU and YRI populations, which likely suggests that association patterns in PD differ among ethnic groups.

Neither rs900147 nor rs2253820 is known to cause a functional change. LD block analysis indicated that rs2253820 is located in a block covering exon 18 of *PER1*, while rs900147 resides in a 24-Kb block containing the promoter region and first exon of *ARNTL*. Therefore, it is possible that functional effects may result from altered transcription or coding sequences. Numerous imputed SNPs also show significant associations with PD. Among these, rs11022743 potentially eliminates the binding site for EVI-1, and rs2279284 adds a putative binding site for CdxA at the promoter region of *ARNTL* (http://genome.ucsc.edu/ENCODE/).

In conclusion, our study demonstrates a significant association between clock genes and sporadic PD in a Chinese population. The association of the *ARNTL* variant (rs900147) was more robust in the TD subtype, while the association of the *PER1* variant (rs2225380) was more robust in the PIGD subtype. Our findings require replication but suggest a causative role for circadian disruption in PD. Targeting circadian disruption and the molecular components of the clock may have therapeutic potential in PD^28^.

## Methods

### Human subjects

PD cases included in the present study were identified from the PD cohort of the Chinese National Consortium on Neurodegenerative Diseases (CNCPD, www.chinapd.cn), established by the Chinese Parkinson Study Group (CPSG), a collaboration of 42 clinical centres managed by the coordination centre at Xuanwu Hospital of Capital Medical University in Beijing. PD was diagnosed by movement disorder specialists using the United Kingdom PD Society Brain Bank Criteria^37^. All subjects were Chinese Han. Patients with a family history of PD or with onset ages under 45 years were excluded from the study. Age, sex and date of disease onset were recorded for all patients. The Unified Parkinson’s Disease Rating Scale (UPDRS) score was recorded for most patients, 1,253 of whom were successfully genotyped. These 1,253 patients were subclassified into three subtypes according to Jankovic’s method^38^. Depending on the dominant motor symptoms, the PD subtype was defined as tremor dominant (TD), postural instability and gait difficulties dominant (PIGD), or intermediate (IND). This classification is based on a ratio obtained by dividing the sum of UPDRS “tremor items” 16, 20 and 21 by the sum of “PIGD items” 13–15, 29 and 30. The TD group consisted of subjects with a ratio ≥1.5. The PIGD group included all patients with a ratio ≤1.0. The remaining subjects were grouped as IND. Healthy adult volunteers, serving as the control group, were matched to cases by age (±5 years), gender, and ethnicity. Individuals with dementia or with family histories of PD in first- or second-degree relatives were excluded from the control group. Informed consent was obtained from all patients and controls. This study was approved by the Xuanwu Hospital ethics committee and complied with national legislation and the International Code of Medical Ethics of the World Medical Association.

### Selection of Tag SNPs

HapMap (Phase II+III) data for Han Chinese (Han Chinese in Bejing, CHB and Han Chinese in Metropolitan Denver, CHD) were used for selecting tagging SNPs in order to match the studied population. Genotypes within the 8 critical clock genes (*NPAS2*, *CLOCK*, *RORB*, *ARNTL*, *CRY1*, *CRY2*, *PER1*, *NR1D1*) and 5-Kb flanking regions were obtained from the HapMap website. Haploview was used for picking up tagging SNPs under pairwise mode with an r^2^ threshold of 0.8. Finally, 132 selected tagging SNPs with minor allele frequencies (MAFs) greater than 0.1 could capture 100% of common SNPs detected in HapMap CHB+CHD populations.

### Genotyping

DNA samples were extracted from venous blood specimens using DNA extraction kits (Tiangen Biotech, Beijing, China). DNA samples (250 ng) were randomly distributed into 96-well plates and sent to the Chinese Academy of Sciences (CAS) key laboratory (Beijing Institute of Genomics, Beijing, China). There, genotypes were determined by laboratory personnel blind to subject status. Genotyping was performed with GoldenGate chips (Illumina, San Diego, CA, USA) and Titanium DNA polymerase according to the manufacturer’s instructions (CloneTech, Mountain View, CA, USA). The GenomeStudio (Illumina, San Diego, CA, USA) genotyping module was employed to call raw data with a genotype call threshold (boundary for calling genotypes relative to its associated cluster) of 0.25. In total, 2,736 (1,394 cases and 1,342 controls) of 2,840 samples were successfully genotyped, with a call rate ≥95%.

### Statistical analysis

Principal component analysis (PCA) was performed on 2,736 individuals with the smartPCA package, as well as multidimensional scaling using an R package^39^. Wigginton’s exact tests were performed for each site among controls to assess whether genotype distributions of each SNP violated Hardy-Weinberg (HW) equilibrium^40^. SNPs with a *P*-value < 0.01 were considered a departure from HW equilibrium and were removed.

Association analysis of the genotype data was conducted with PLINK (v1.07)^41^. Bonferroni adjustment was implemented for multiple tests. For association analysis, Cochran-Armitage trend models were used to study the association between each SNP and PD. Age- and sex-adjusted logistic regression was also tested assuming an additive genetic model. The odds ratio (OR) was calculated using the Cochran-Armitage trend test with the ancestral allele (determined from the chimpanzee sequence) for reference. Pairwise epistasis tests were also performed to evaluate the interaction between different genetic loci using proportions significance of valid tests for multiple tests correction. The linkage disequilibrium (LD) blocks were constructed using Haploview v4.2^42^. Haplotype-based association analysis was performed on determined blocks, and the permutation method (10,000 permutations) was used to obtain the empirical *P*-value. The regional association plots and linkage disequilibrium plots were performed with SNAP (SNP Annotation and Proxy Search, http://www.broadinstitute.org/mpg/snap/ldplot.php).

Pre-phasing the haplotype was performed using the SHAPEIT algorithm due to its lower error rate than other software^43^. Prediction of untyped SNPs was carried out using IMPUTE2^44^ based on the 1,000 Genomes phase 1 integrated variant set (b37; December 2013). Imputed data were analysed using SNPTEST v2^45^ to account for uncertainties in SNP prediction. We used a strict cut-off (0.85) that provided an allelic dosage *R*^2^ correlation between real and imputed genotypes greater than 0.8, and it showed an optimal balance between accuracy and power.

We used the following tracks, implemented in the University of California, Santa Cruz, Genome Browser, to predict the putative promoter regions of *ARNTL* and *PER1*: ENCODE Transcription Factor ChIP-Seq, CpG Islands, and ENCODE Promoter-associated Histone Mark. To analyse the possible effect of significant SNPs on putative transcription factor binding sites, 100-bp sequences surrounding SNPs were analysed using TFSEARCH (http://www.cbrc.jp/research/db/TFSEARCH.html). The parameters used for prediction were vertebrate classification only, with a threshold score of 85.0 points.

A genetic power calculator^46^ was used for power calculations. With the sample size of this study, the power of detecting a significant association can reach 0.85, with a genotype relative risk of 1.3 and high risk allele frequency of 0.15.

## Additional Information

**How to cite this article**: Gu, Z. *et al.* Association of *ARNTL* and *PER1* genes with Parkinson's disease: a case-control study of Han Chinese. *Sci. Rep.* **5**, 15891; doi: 10.1038/srep15891 (2015).

## Supplementary Material

Supplementary Table 1

## Figures and Tables

**Table 1:** Demographic and clinical characteristics of the participants with genotyping data.

Characteristics	Controls (n = 1342)	PD cases (n = 1394)	PD cases with UPDRS (n = 1253)
Gender, n (%)			
Male	798 (59.5)	830 (59.5)	749 (59.8)
Female	544 (40.5)	564 (40.5)	504 (40.2)
Age at collection (mean ± SD)	66.5 ± 7.2	65.2 ± 8.4	65.2 ± 8.4
Age of onset (mean ± SD)	—	60.4 ± 8.5	60.3 ± 8.5

PD, Parkinson’s disease; UPDRS, unified Parkinson's disease rating scale.

**Table 2:** Association analysis between SNPs and Parkinson’s disease.

Gene	SNP^a^	allele A>B^b^	case	control	MAF	*P*-value		OR (95% CI)^e^
			AA/AB/BB^b^	AA/AB/BB^b^		Trend^c^ (bonferroni^f^)	LR^d^ (bonferroni^f^)	
*NPAS2*	rs930309	T>A	307/647/425	226/661/444	0.438	4.07 × 10^−3^	6.18 × 10^−3^	0.85 (0.77–0.95)
*NPAS2*	rs12479086	G>A	1031/315/37	1049/268/15	0.127	1.67 × 10^−3^	1.51 × 10^−3^	1.30 (1.11–1.53)
*CLOCK*	rs3749474	G>A	195/663/535	165/603/569	0.364	2.56 × 10^−2^	2.88 × 10^−2^	0.88 (0.79–0.98)
*RORB*	rs17611535	G>A	1140/237/14	1042/273/24	0.107	3.72 × 10^−3^	4.02 × 10^−3^	0.77 (0.65–0.92)
*ARNTL*	rs900147	G>A	289/715/369	233/612/455	0.444	3.33 × 10^−5^ (4.23 × 10^−3^)	5.13 × 10^−5^ (6.52 × 10^−3^)	0.80 (0.71–0.89)
*ARNTL*	rs7950226	G>A	447/704/238	493/639/198	0.407	8.84 × 10^−3^	6.84 × 10^−3^	0.86 (0.77–0.96)
*ARNTL*	rs11605776	C>A	365/675/349	290/667/377	0.487	4.91 × 10^−3^	6.02 × 10^−3^	0.86 (0.77–0.95)
*ARNTL*	rs10832022	A>G	405/683/299	435/666/232	0.443	4.77 × 10^−3^	7.16 × 10^−3^	1.17 (1.05–1.30)
*ARNTL*	rs11022765	C>A	501/667/222	541/615/175	0.382	4.85 × 10^−3^	6.99 × 10^−3^	1.17 (1.05–1.31)
*ARNTL*	rs1562437	G>A	39/421/926	52/463/809	0.197	1.33 × 10^−3^	1.36 × 10^−3^	0.81 (0.70–0.92)
*PER1*	rs2253820	A>G	630/632/121	711/501/88	0.289	5.30 × 10^−6^ (6.73 × 10^−4^)	1.37 × 10^−5^ (1.74 × 10^−3^)	1.31 (1.17–1.48)
*NR1D1*	rs3744805	G>A	231/610/512	245/646/409	0.416	2.94 × 10^−3^	2.43 × 10^−3^	1.18 (1.06–1.32)

SNP, single nucleotide polymorphism; MAF, minor allele frequency; OR, odds ratio; CI, confidence interval, LR, logistic regression.

^a^Only SNPs with a *P*-value < 0.01 under the trend model are shown.

^b^A allele is ancestral allele and B allele is alternative allele.

^c^Asymptotic *P*-value.

^d^With age and sex as covariates.

^e^Odds ratio under Cochran-Armitage trend model with 95% confidence interval.

^f^Bonferroni *P*-value (*P*-value × 125 tests).

**Table 3:** Association analysis between the *ARNTL* and *PER1* genes and the TD and PIGD subtypes of PD.

Gene	SNP^a^	*P*-values (Trend model)		OR (95% CI)^b^	
		TD (Bonferroni)	PIGD (Bonferroni)	TD	PIGD
*ARNTL*	rs900147	6.53 × 10^−2^	3.22 × 10^−4^ (0.0415)	0.86(0.73–1.01)	0.78(0.68–0.89)
*ARNTL*	rs7950226	4.48 × 10^−2^	1.40 × 10^−2^	0.85(0.72–1.00)	0.85(0.74–0.97)
*ARNTL*	rs11605776	1.13 × 10^−1^	1.89 × 10^−2^	0.88(0.75–1.03)	0.85(0.75–0.97)
*ARNTL*	rs10832022	9.52 × 10^−2^	1.59 × 10^−2^	1.14(0.98–1.34)	1.18(1.03–1.35)
*ARNTL*	rs11022765	2.22 × 10^−1^	8.05 × 10^−3^	1.11(0.94–1.30)	1.20(1.05–1.38)
*ARNTL*	rs7941761	5.17 × 10^−1^	6.76 × 10^−3^	1.05(0.90–1.24)	1.20(1.05–1.37)
*ARNTL*	rs1562437	1.99 × 10^−2^	2.50 × 10^−3^	0.79(0.64–0.97)	0.77(0.65–0.92)
*PER1*	rs2253820	6.03 × 10^−5^ (0.00753)	2.17 × 10^−3^	1.42(1.19–1.68)	1.26(1.08–1.45)

OR(95% CI), Odds ratio (95% confidence interval).

^a^SNPs with *P*-value less than 0.01.

^b^Odds ratio under Cochran-Armitage trend model with chimpanzee allele as reference allele.

**Table 4:** Association of Haplotypes with PD.

SNPs in LD block	Haplotype^a^	Haplotype frequency		*P*-value	permutation *P*-value
		Case	Control		
*ARNTL*					
rs900147	AAACG	0.377	0.419	0.017	0.018
rs11605776	GCGAA	0.248	0.210	9.00 × 10^−4^	0.010
rs10832022	ACGAA	0.102	0.111	0.258	0.976
rs11022765	GAACG	0.073	0.073	0.940	1.000
rs7941761	GCACG	0.061	0.059	0.804	0.447
*PER1*					
rs2225380	AA	0.462	0.522	7.53 × 10^−6^	1.00 × 10^−4^
rs2304911	GA	0.314	0.259	8.32 × 10^−6^	1.00 × 10^−4^
	AG	0.222	0.216	0.571	0.992

LD, linkage disequilibrium.

^a^Haplotypes with an allele frequency greater than 0.05 were listed.
