# Supplementary material for: Association of ARNTL and PER1 genes with Parkinson's disease: a case-control study of Han Chinese
Source: Sci Rep. 2015 Oct 28;5:15891. doi: 10.1038/srep15891 (PMC4623766; doi:10.1038/srep15891)
Supplement: Supplementary Table 1 [file srep15891-s1.doc]

**Supplementary information**

**Association of *ARNTL* and *PER1* genes with Parkinson's disease: a case-control study of Han Chinese**

Zhuqin Gu^1,§^, BinBin Wang^2,3,§^, Yong-Biao Zhang^2,§,*^, Hui Ding^1^, Yanli Zhang^4^, Jun Yu^2,3^, Mingliang Gu^2^, Piu Chan^1,4^, Yanning Cai^1,4,*^

^1^Department of Neurobiology, Xuanwu Hospital of Capital Medical University, Key Laboratory for Neurodegenerative Diseases of the Ministry of Education, Beijing 100053, P.R. China

^2^CAS Key Laboratory of Genome Sciences and Information, Beijing Institute of Genomics, Chinese Academy of Sciences, Beijing 100101, P.R. China

^3^National Research Institute for Family Planning, Beijing 100081, P.R. China

^4^Department of Neurology, Xuanwu Hospital of Capital Medical University, Key Laboratory for Neurodegenerative Diseases of the Ministry of Education, Beijing 100053, P.R. China

^§^These authors contributed equally to this work.

Running title: Clock genes and Parkinson’s disease

**Declaration:** There is no conflict of interest among the authors.

^*^Corresponding authors: Yanning Cai, Ph.D., Department of Neurobiology, Xuanwu Hospital of Capital Medical University, 45 Changchun Street, Beijing 100053, China. Tel: +86 (10) 83198266; Fax: +86 (10) 83198266; E-mail: [Yanningcai@yahoo.com](mailto:Yanningcai@yahoo.com);

Or Yong-Biao Zhang, Ph.D., CAS Key Laboratory of Genome Sciences and Information, Beijing Institute of Genomics, Chinese Academy of Sciences, 1 Beichen West Road, Beijing 100101, China. Tel: +86 (10) 80497586; Fax: +86 (10) 80497720; E-mail: [zhangyongbiao@gmail.com](mailto:zhangyongbiao@gmail.com)

Supplementary Table 1 Association results of all 125 SNPs under Trend model

| Gene | SNP^a^ | allele A > B | MAF^b^ | | *P*-value | OR (95% CI)^c^ |
| --- | --- | --- | --- | --- | --- | --- |
|  |  |  | Case | Control |  |  |
| NPAS2 | rs12475500 | A>G | 0.2046 | 0.193 | 0.2804 | 1.076 (0.9413 - 1.229) |
| NPAS2 | rs10172146 | G>A | 0.3938 | 0.3964 | 0.8439 | 0.9892 (0.8874 - 1.103) |
| NPAS2 | rs6542992 | A>C | 0.4982 | 0.4981 | 0.9958 | 1 (0.899 - 1.113) |
| NPAS2 | rs4349369 | G>A | 0.3418 | 0.3678 | 0.07756 | 0.8929 (0.7871 - 1.013) |
| NPAS2 | rs3860456 | T>A | 0.2649 | 0.2823 | 0.1449 | 0.9161 (0.8127 - 1.033) |
| NPAS2 | rs6542994 | A>G | 0.406 | 0.4057 | 0.9808 | 1.001 (0.8985 - 1.116) |
| NPAS2 | rs10193688 | A>G | 0.2826 | 0.2863 | 0.7625 | 0.9819 (0.8701 - 1.108) |
| NPAS2 | rs1561002 | G>A | 0.264 | 0.2547 | 0.4337 | 1.05 (0.9299 - 1.185) |
| NPAS2 | rs7598826 | A>G | 0.3765 | 0.3469 | 0.02258 | 1.137 (1.018 - 1.27) |
| NPAS2 | rs1811399 | C>A | 0.357 | 0.3407 | 0.2105 | 1.074 (0.9605 - 1.201) |
| NPAS2 | rs2043534 | A>G | 0.234 | 0.2553 | 0.07131 | 0.8912 (0.7875 - 1.009) |
| NPAS2 | rs930309 | T>A | 0.4572 | 0.4181 | 0.004065 | 1.172 (1.053 - 1.305) |
| NPAS2 | rs41349646 | G>A | 0.324 | 0.3475 | 0.06268 | 0.9 (0.8041 - 1.007) |
| NPAS2 | rs12472321 | A>G | 0.3586 | 0.3554 | 0.8215 | 1.014 (0.9015 - 1.141) |
| NPAS2 | rs13407950 | G>A | 0.2941 | 0.2971 | 0.8025 | 0.9856 (0.877 - 1.108) |
| NPAS2 | rs17024926 | A>G | 0.4726 | 0.4917 | 0.154 | 0.9262 (0.8326 - 1.03) |
| NPAS2 | rs6740935 | A>G | 0.1739 | 0.2034 | 0.005093 | 0.8246 (0.719 - 0.9458) |
| NPAS2 | rs12712084 | A>G | 0.3906 | 0.3902 | 0.9791 | 1.001 (0.898 - 1.117) |
| NPAS2 | rs1369481 | A>G | 0.3023 | 0.2778 | 0.05777 | 1.127 (0.9967 - 1.273) |
| NPAS2 | rs7585886 | A>G | 0.1341 | 0.1069 | 0.002539 | 1.294 (1.098 - 1.525) |
| NPAS2 | rs12479086 | A>G | 0.1406 | 0.1119 | 0.001666 | 1.299 (1.106 - 1.527) |
| NPAS2 | rs11674168 | A>G | 0.1964 | 0.2104 | 0.1979 | 0.9174 (0.8039 - 1.047) |
| NPAS2 | rs6729727 | C>A | 0.276 | 0.2954 | 0.1128 | 0.9094 (0.8083 - 1.023) |
| NPAS2 | rs11894322 | C>A | 0.2265 | 0.2068 | 0.07948 | 1.123 (0.9872 - 1.278) |
| NPAS2 | rs6725296 | A>G | 0.1585 | 0.1553 | 0.7406 | 1.025 (0.8856 - 1.186) |
| NPAS2 | rs356647 | A>G | 0.304 | 0.302 | 0.8754 | 1.009 (0.899 - 1.133) |
| NPAS2 | rs3820785 | G>A | 0.3364 | 0.3316 | 0.7055 | 1.022 (0.9133 - 1.144) |
| NPAS2 | rs3754674 | C>G | 0.2703 | 0.2691 | 0.9229 | 1.006 (0.8925 - 1.134) |
| NPAS2 | rs17025005 | A>G | 0.2334 | 0.2298 | 0.7578 | 1.02 (0.8994 - 1.158) |
| NPAS2 | rs7605570 | A>G | 0.4215 | 0.4353 | 0.304 | 0.9449 (0.8486 - 1.052) |
| NPAS2 | rs3739005 | A>G | 0.4816 | 0.469 | 0.3524 | 1.052 (0.9455 - 1.17) |
| NPAS2 | rs3820787 | G>A | 0.4415 | 0.4502 | 0.5207 | 0.9651 (0.8674 - 1.074) |
| NPAS2 | rs12612424 | A>C | 0.3711 | 0.3514 | 0.1354 | 1.089 (0.9751 - 1.217) |
| NPAS2 | rs876060 | A>T | 0.316 | 0.3522 | 0.005019 | 0.8496 (0.7572 - 0.9534) |
| NPAS2 | rs12622050 | G>A | 0.3727 | 0.3929 | 0.127 | 0.918 (0.823 - 1.024) |
| NPAS2 | rs17025078 | A>G | 0.3003 | 0.3098 | 0.4414 | 0.9562 (0.8521 - 1.073) |
| NPAS2 | rs2305159 | A>C | 0.2536 | 0.2646 | 0.3509 | 0.944 (0.8363 - 1.066) |
| NPAS2 | rs9679638 | G>A | 0.2822 | 0.2758 | 0.5971 | 1.032 (0.917 - 1.162) |
| NPAS2 | rs1542179 | A>G | 0.2834 | 0.3023 | 0.1236 | 0.9128 (0.812 - 1.026) |
| NPAS2 | rs1542178 | A>G | 0.2228 | 0.2334 | 0.3513 | 0.9414 (0.8296 - 1.068) |
| NPAS2 | rs3768988 | G>A | 0.3283 | 0.2971 | 0.01285 | 1.156 (1.031 - 1.297) |
| NPAS2 | rs2278728 | A>G | 0.2478 | 0.2609 | 0.2731 | 0.9336 (0.8264 - 1.055) |
| NPAS2 | rs2278727 | A>G | 0.3329 | 0.3259 | 0.5865 | 1.032 (0.9213 - 1.155) |
| NPAS2 | rs6719533 | G>A | 0.4752 | 0.4722 | 0.8238 | 1.012 (0.91 - 1.126) |
| NPAS2 | rs3768994 | G>A | 0.09251 | 0.08738 | 0.5113 | 1.065 (0.8844 - 1.282) |
| NPAS2 | rs935401 | T>A | 0.2302 | 0.2494 | 0.09468 | 0.8997 (0.7945 - 1.019) |
| NPAS2 | rs1374324 | G>A | 0.4252 | 0.4489 | 0.0774 | 0.9081 (0.8157 - 1.011) |
| NPAS2 | rs11690047 | C>G | 0.1603 | 0.1743 | 0.1586 | 0.904 (0.7838 - 1.043) |
| NPAS2 | rs2278721 | C>G | 0.2462 | 0.2335 | 0.2749 | 1.072 (0.9464 - 1.214) |
| NPAS2 | rs3739011 | G>A | 0.1004 | 0.09172 | 0.2775 | 1.106 (0.9233 - 1.324) |
| NPAS2 | rs1302 | A>C | 0.2516 | 0.2284 | 0.04147 | 1.136 (1.003 - 1.287) |
| NPAS2 | rs17719821 | C>G | 0.1579 | 0.1783 | 0.04569 | 0.8642 (0.7496 - 0.9963) |
| NPAS2 | rs12474501 | A>C | 0.3968 | 0.3805 | 0.2197 | 1.071 (0.9601 - 1.195) |
| NPAS2 | rs2289953 | G>A | 0.3466 | 0.3605 | 0.3114 | 0.9413 (0.8368 - 1.059) |
| CLOCK | rs1048004 | A>C | 0.08472 | 0.07822 | 0.388 | 1.091 (0.8969 - 1.326) |
| CLOCK | rs3749474 | G>A | 0.378 | 0.3489 | 0.02559 | 1.134 (1.015 - 1.266) |
| CLOCK | rs10002541 | G>A | 0.2999 | 0.2772 | 0.06299 | 1.117 (0.9937 - 1.256) |
| CLOCK | rs1979605 | A>G | 0.08249 | 0.1007 | 0.02436 | 0.8029 (0.6663 - 0.9675) |
| RORB | rs10869410 | A>G | 0.1264 | 0.1371 | 0.2459 | 0.9109 (0.778 - 1.066) |
| RORB | rs10491929 | G>A | 0.1498 | 0.1723 | 0.02636 | 0.8464 (0.7324 - 0.9781) |
| RORB | rs10869412 | G>A | 0.2261 | 0.2562 | 0.009665 | 0.848 (0.7488 - 0.9604) |
| RORB | rs17611535 | A>G | 0.09526 | 0.1199 | 0.003721 | 0.7731 (0.6508 - 0.9182) |
| RORB | rs11144003 | A>T | 0.09424 | 0.09019 | 0.6084 | 1.05 (0.8735 - 1.261) |
| RORB | rs17612113 | C>A | 0.09532 | 0.1155 | 0.01634 | 0.8069 (0.6782 - 0.96) |
| RORB | rs17060365 | G>A | 0.07866 | 0.07704 | 0.8259 | 1.023 (0.8391 - 1.247) |
| RORB | rs10869430 | G>A | 0.4131 | 0.4041 | 0.502 | 1.038 (0.9311 - 1.157) |
| RORB | rs1831121 | G>A | 0.2583 | 0.2509 | 0.5382 | 1.039 (0.9201 - 1.174) |
| RORB | rs11144029 | G>A | 0.1964 | 0.1914 | 0.6473 | 1.032 (0.9022 - 1.181) |
| RORB | rs3750420 | G>A | 0.4266 | 0.4505 | 0.07391 | 0.9075 (0.815 - 1.01) |
| RORB | rs1013078 | A>T | 0.3065 | 0.3042 | 0.85 | 1.011 (0.9007 - 1.135) |
| RORB | rs10512037 | G>A | 0.4528 | 0.428 | 0.06207 | 1.106 (0.9933 - 1.231) |
| RORB | rs11144033 | G>A | 0.1363 | 0.1271 | 0.3115 | 1.084 (0.9265 - 1.269) |
| RORB | rs968357 | G>A | 0.13 | 0.1441 | 0.1316 | 0.8875 (0.76 - 1.036) |
| RORB | rs11144043 | G>C | 0.1778 | 0.1822 | 0.6754 | 0.9707 (0.8452 - 1.115) |
| RORB | rs10781247 | G>A | 0.4388 | 0.4126 | 0.05084 | 1.113 (0.9997 - 1.24) |
| RORB | rs7021908 | A>G | 0.1462 | 0.1529 | 0.4851 | 0.9487 (0.8174 - 1.101) |
| RORB | rs11144045 | C>A | 0.3181 | 0.2998 | 0.1404 | 1.09 (0.9709 - 1.223) |
| RORB | rs7865407 | C>A | 0.09884 | 0.09204 | 0.3972 | 1.082 (0.9026 - 1.297) |
| RORB | rs7033059 | G>A | 0.317 | 0.307 | 0.4252 | 1.048 (0.9341 - 1.175) |
| RORB | rs11144053 | G>C | 0.1221 | 0.1114 | 0.2226 | 1.11 (0.9401 - 1.31) |
| RORB | rs10521463 | C>A | 0.3296 | 0.3686 | 0.006796 | 0.8423 (0.7422 - 0.9559) |
| RORB | rs17060410 | G>A | 0.2915 | 0.3084 | 0.1813 | 0.9228 (0.8206 - 1.038) |
| RORB | rs1410228 | G>A | 0.3236 | 0.3391 | 0.2171 | 0.9323 (0.8327 - 1.044) |
| RORB | rs943632 | G>A | 0.4493 | 0.4338 | 0.2384 | 1.065 (0.9567 - 1.185) |
| RORB | rs3750424 | G>A | 0.2486 | 0.2588 | 0.38 | 0.9472 (0.8382 - 1.07) |
| RORB | rs34369393 | A>G | 0.2792 | 0.2924 | 0.2817 | 0.9371 (0.8321 - 1.055) |
| RORB | rs900147 | G>A | 0.4709 | 0.4146 | 3.33E-05 | 1.256 (1.128 - 1.4) |
| ARNTL | rs7950226 | G>A | 0.4248 | 0.3891 | 0.006782 | 1.159 (1.04 - 1.292) |
| ARNTL | rs11605776 | C>A | 0.5058 | 0.4674 | 0.004913 | 1.166 (1.048 - 1.297) |
| ARNTL | rs7130064 | A>G | 0.1736 | 0.1776 | 0.6918 | 0.9725 (0.8445 - 1.12) |
| ARNTL | rs4757144 | A>G | 0.3973 | 0.4105 | 0.3274 | 0.9465 (0.8494 - 1.055) |
| ARNTL | rs10832022 | G>A | 0.4618 | 0.4239 | 0.004768 | 1.166 (1.048 - 1.298) |
| ARNTL | rs11022765 | A>C | 0.3996 | 0.3625 | 0.004854 | 1.171 (1.049 - 1.306) |
| ARNTL | rs10766077 | G>A | 0.3448 | 0.3527 | 0.5395 | 0.9659 (0.8636 - 1.08) |
| ARNTL | rs7941761 | A>G | 0.4474 | 0.4165 | 0.01968 | 1.134 (1.018 - 1.263) |
| ARNTL | rs2896635 | A>T | 0.3519 | 0.346 | 0.6504 | 1.026 (0.918 - 1.147) |
| ARNTL | rs1562437 | A>G | 0.18 | 0.2141 | 0.001326 | 0.8057 (0.7045 - 0.9215) |
| ARNTL | rs1026070 | C>G | 0.1975 | 0.1936 | 0.7157 | 1.025 (0.8965 - 1.172) |
| ARNTL | rs7126303 | A>G | 0.1462 | 0.1638 | 0.07316 | 0.874 (0.7536 - 1.014) |
| ARNTL | rs11022776 | G>C | 0.08537 | 0.09485 | 0.2186 | 0.8907 (0.74 - 1.072) |
| ARNTL | rs2290036 | G>A | 0.1598 | 0.163 | 0.7471 | 0.9764 (0.8451 - 1.128) |
| ARNTL | rs1868049 | G>A | 0.4769 | 0.4844 | 0.5854 | 0.9705 (0.8722 - 1.08) |
| ARNTL | rs3789327 | G>A | 0.3129 | 0.3089 | 0.7474 | 1.019 (0.9086 - 1.143) |
| ARNTL | rs11022778 | C>A | 0.1369 | 0.1378 | 0.9225 | 0.9924 (0.85 - 1.159) |
| ARNTL | rs3816358 | A>C | 0.1739 | 0.1513 | 0.0275 | 1.181 (1.02 - 1.368) |
| ARNTL | rs11022780 | A>G | 0.2762 | 0.2701 | 0.6115 | 1.031 (0.9154 - 1.162) |
| CRY2 | rs11022785 | G>A | 0.475 | 0.4707 | 0.7493 | 1.017 (0.9142 - 1.132) |
| CRY2 | rs11022789 | A>G | 0.1818 | 0.1765 | 0.6046 | 1.037 (0.9024 - 1.191) |
| CRY2 | rs6485646 | A>G | 0.2493 | 0.2383 | 0.3508 | 1.061 (0.9371 - 1.202) |
| CRY2 | rs4755345 | G>A | 0.46 | 0.4568 | 0.8148 | 1.013 (0.9104 - 1.127) |
| CRY2 | rs2292910 | C>A | 0.3128 | 0.3069 | 0.6412 | 1.028 (0.9165 - 1.153) |
| CRY2 | rs3824872 | C>A | 0.4009 | 0.3883 | 0.3458 | 1.054 (0.9453 - 1.175) |
| CRY2 | rs1554338 | G>A | 0.08309 | 0.09467 | 0.1255 | 0.8667 (0.7187 - 1.045) |
| CRY1 | rs11113153 | A>G | 0.2962 | 0.3016 | 0.6642 | 0.9746 (0.8677 - 1.095) |
| CRY1 | rs3741892 | C>G | 0.259 | 0.2366 | 0.05287 | 1.128 (0.9968 - 1.276) |
| CRY1 | rs11113179 | A>G | 0.1356 | 0.1477 | 0.202 | 0.9054 (0.7773 - 1.055) |
| CRY1 | rs11113181 | G>A | 0.2879 | 0.293 | 0.6717 | 0.9753 (0.8676 - 1.096) |
| PER1 | rs9914077 | G>A | 0.09668 | 0.1069 | 0.2083 | 0.8942 (0.7496 - 1.067) |
| PER1 | rs2253820 | G>A | 0.316 | 0.2604 | 5.30E-06 | 1.312 (1.165 - 1.478) |
| PER1 | rs2304911 | G>A | 0.226 | 0.218 | 0.4755 | 1.047 (0.9203 - 1.191) |
| PER1 | rs3027178 | C>A | 0.2793 | 0.2748 | 0.7033 | 1.023 (0.9081 - 1.152) |
| NR1D1 | rs3744805 | G>A | 0.3962 | 0.4369 | 0.002944 | 0.8455 (0.758 - 0.9431) |
| NR1D1 | rs2071427 | A>G | 0.4849 | 0.4797 | 0.6936 | 1.021 (0.9178 - 1.137) |
| NR1D1 | rs2269457 | G>A | 0.4716 | 0.4792 | 0.5636 | 0.9701 (0.8717 - 1.08) |
| NR1D1 | rs12941497 | A>G | 0.4616 | 0.4785 | 0.2075 | 0.9343 (0.8397 - 1.04) |

a: rs ID of each SNP

b: Minor allele frequency

c: Odds ratio under Cochran-Armitage trend model with 95% confident interval for major allele.
